# Editorial: A decade after the collapse: Financial crises, austerity, and their responses

**DOI:** 10.3389/fsoc.2022.993223

**Published:** 2022-08-10

**Authors:** Maximilian Conrad, Helga Kristín Hallgrímsdóttir, Edwin Hodge

**Affiliations:** ^1^Faculty of Political Science, University of Iceland, Reykjavík, Iceland; ^2^School of Public Administration, University of Victoria, Victoria, BC, Canada

**Keywords:** austerity, crisis, financial crisis 2007–2009, populism, contentious politics, status anxiety

Critical junctures in history are often preceded by events that can be interpreted, in hindsight, as clear signs of an imminent crisis. However, in the perception of wider publics, the beginning of a crisis is often connected to more specific moments at which, it seems, a crisis has arrived *all of a sudden*. Such moments tend to become deeply engrained in the collective memory of a nation. In Icelandic collective memory, no event has characterized the imminent global financial crisis as dramatically as a televised speech by then Prime Minister Geir Haarde on October 6, 2008. In this speech, Haarde informed his fellow Icelanders about the critical state of the country's three major banks, all of which ended up going bankrupt within days, marking the beginning of the Icelandic *kreppa* (or crash). Most Icelanders today remember the speech for the words with which Haarde ended it: God bless Iceland.

A decade and a half after it began in North America, the global financial crisis may seem a distant memory by now, all the more so given the serious challenges facing the international community today. But the policies adopted in the wake of the global financial crisis have evidently had a lasting impact on the affected countries, but also for supranational polities such as the European Union. On the surface, the kinds of austerity measures that were adopted by national governments and/or imposed by supranational actors (such as the “troika,” composed of the European Commission, the European Central Bank and the International Monetary Fund) resulted in a drastic reduction of public spending and structural reforms for the sake of stimulating stagnant national economies. But such measures have obviously had much more far-reaching implications. At the level of the nation state, as some of the contributions in this Research Topic underline, this approach resulted in the rise of radical social and political movements on both sides of the political spectrum as well as on both sides of the Atlantic. As a result, these austerity policies have had a lasting impact on party politics in Europe and North America, characterized by increasing polarization and the emergence of new political parties. In the context of European integration, furthermore, austerity policies have undermined a sense of European collective identity by deepening stereotypes of rich Northern and poor Southern member states.

Five of the six contributions in this Research Topic study the impact of austerity policies on various political and social processes in Europe and North America. Specifically, they highlight the role that austerity policies have played as a significant driving force in the resurgence of populism and/or nationalism in Europe and North America. Hallgrímsdóttir et al. analyze the role of “crisis narratives” and “austerity talk” as devices that have facilitated the emergence and/or resurgence of right-wing populist parties in France, Germany and the United Kingdom. Their analysis underscores how right-wing actors such as the French *Rassemblement National*, the German *Alternative für Deutschland* and the British *UK Independence Party* have used perceptions of crisis as narrative devices to advance their respective political agendas, frequently in ways that intersect with memory politics and “‘forgetful' renderings of the past” that pit citizens against non-citizens as well as deserving against non-deserving migrants.

The German AfD takes center stage in the contributions by Schmidtke and Conrad. For Conrad, the emergence of the AfD is the clearest and politically most relevant result of the Eurozone debt crisis in Germany. Using a process-tracing design, this contribution highlights the emergence and development of the AfD from a soft Euroskeptic party into a typical right-wing populist party, evidenced both by increasingly radical leaderships and a thematic shift away from the common currency toward issues of migration and multiculturalism. Schmidtke's contribution looks at the global financial crisis and the resulting austerity policies as the root cause of populist, anti-establishment protest on both extremes of the political spectrum in Germany. For Schmidtke, the consequence of austerity and social inequality in Germany is not merely the rise of the AfD on the right of the political spectrum, but also the emergence of the *Stand Up* movement as a corresponding development on the left of the political spectrum.

Finnsdóttir studies the reasons for the emergence of radical national parties, but takes a broader quantitative and comparative perspective on the countries of Central and Eastern Europe. Specifically, she analyzes the role of austerity policies in Central and Eastern Europe as a key role as a driver of labor emigration that has created the “key pathway” to the emergence of a strong radical right in the analyzed countries.

Although it is the only contribution in this Research Topic that focuses on the North American context, Hodge's analysis of the rebirth of the Sovereign Citizen Movement in the United States and Canada clearly strikes a chord with the other contributions in this Research Topic. Hodge interprets this rebirth as an act of resistance resulting from austerity policies. Similar to the contributions by Schmidtke, Finnsdóttir and Hallgrímsdóttir, Hodge emphasizes the importance of “status anxiety and uncertainty.” In the case of the Sovereign Citizen Movement, such anxieties are fueled also by conspiratorial thinking and result in a radical concept of citizenship that stands in direct conflict with the state.

In the last contribution to this Research Topic, Ólafsson offers a somewhat different take on the theme of the global financial crisis. Instead of addressing the causal impact of austerity policies, Ólafsson addresses the way in which the question of political and legal accountability for the crisis was handled in the court case against Iceland's former Prime Minister Geir Haarde. Specifically, Ólafsson presents a critical discussion of Prime Minister Geir Haarde's conviction for negligence to consult with his ministers on measures to prepare for the coming crisis. This conviction, Ólafsson argues, was largely based on epistemic grounds: it was considered impossible for Haarde *not* to have understood, well before delivering his “God bless Iceland” speech on October 6, the severity of the threat the country was facing at the time.

Though each of these contributions focused on the immediate after-effects of the 2007 collapse, they remain salient even today, as states around the world continue to grapple with the effects of the COVID-19 epidemic. Just as the financial collapse spurred the rise of populist and conspiratorial movements—to say nothing of the economic impacts—the social, political, and economic upheavals of the pandemic have fostered the rise of new populist movements who appear to have laid their foundations in those earlier footprints. The contributions of this Research Topic are therefore more than retrospectives: they also offer a glimpse into the present and the future.

## Author contributions

All authors listed have made a substantial, direct, and intellectual contribution to the work and approved it for publication.

## Funding

This study was supported by SSRHC Insight Grant 435-2016-0642.

## Conflict of interest

The authors declare that the research was conducted in the absence of any commercial or financial relationships that could be construed as a potential conflict of interest.

## Publisher's note

All claims expressed in this article are solely those of the authors and do not necessarily represent those of their affiliated organizations, or those of the publisher, the editors and the reviewers. Any product that may be evaluated in this article, or claim that may be made by its manufacturer, is not guaranteed or endorsed by the publisher.

